# Public Dental Workforce Capacity and Inequality in Spain

**DOI:** 10.1016/j.identj.2026.109687

**Published:** 2026-06-18

**Authors:** Manuel Jesús Enciso-Ripoll, José Enrique Iranzo-Cortés, Manuel Bravo, Francisco Javier Silvestre

**Affiliations:** a Department of Dentistry, School of Health Sciences, Universidad Cardenal Herrera-CEU, CEU Universities, Valencia, Spain; b Dental Primary Care, València Arnau de Vilanova-Lliria Public Health Department, Conselleria de Sanitat, Valencia, Spain; c Department of Stomatology, Faculty of Medicine and Dentistry, Universitat de València, Valencia, Spain; d Department of Stomatology, Faculty of Dentistry, Universidad de Granada, Granada, Spain

**Keywords:** Dental workforce, Oral health policy, Public oral health, Workforce planning, Spain, Health equity

## Abstract

**Introduction and aims:**

Spain combines one of Europe's highest dentist densities with limited public oral health coverage and marked regional variation in publicly funded provision. This study quantified total, salaried public and privately accredited dental workforce capacity across Spain and assessed implications for public oral health system planning and equity.

**Methods:**

A cross-sectional national workforce analysis was conducted across the 17 Autonomous Communities and the Autonomous Cities of Ceuta and Melilla. Data were obtained through formal transparency requests to all administrations, supplemented by official professional registration data, population statistics and Ministry of Health records. Indicators included total and salaried public dentists per 100,000 inhabitants, population load per public dentist, the public/total ratio and a Structural Dependence Index (SDI) quantifying reliance on accredited private providers.

**Results:**

In 2024-2025, Spain registered 42,860 active dentists (84.68/100,000) but only 1685 salaried public dentists (mean territorial density: 3.36/100,000; mean territorial load: 31,590 inhabitants per public dentist). The public/total ratio ranged from 2.0% in Madrid to 7.6% in Castilla-La Mancha. A further 6810 privately accredited providers participated in publicly funded programmes across 11 mixed-model regions, yielding an effective public-system workforce of 8495 professionals (17.21/100,000). Among territories with reported individual accredited-provider counts, SDI ranged from 0% in direct public-provision regions to 95.4% in País Vasco; ten of eleven mixed-model regions recorded SDI values above 80%.

**Conclusion:**

Spain does not face a shortage of dentists, but a structural imbalance between abundant private professional supply and limited, unevenly distributed public-system capacity.

**Clinical Relevance:**

High aggregate dentist density is a poor proxy for public oral health system capacity. Workforce planning standards and stronger monitoring systems are needed to translate expanded publicly funded oral health coverage into effective and equitable access.

## Introduction

Dental caries and periodontal disease are among the most prevalent chronic conditions globally, generating substantial pain, functional impairment and productivity losses. Their economic burden extends beyond direct treatment costs to include absenteeism and reduced work capacity, falling disproportionately on socioeconomically disadvantaged populations.^1^ The systemic consequences of oral disease further underscore its status as a primary health care priority: periodontal disease is independently associated with type 2 diabetes in a bidirectional relationship,^2^ cardiovascular disease and adverse pregnancy outcomes,^3^, ^4^ and emerging evidence implicates *Porphyromonas gingivalis* in the pathogenesis of Alzheimer's disease.^5^ These associations transform oral health from a dental specialty into a primary care concern whose exclusion from universal coverage generates costs.^1^^,^^6^

Spain ranks among the world's healthiest nations,^7^ yet public oral health coverage within the National Health System (SNHS) has historically been confined to a limited set of interventions under Royal Decree 1030/2006,^8^ most recently expanded, though still partially, by Order SND/606/2024.^9^ Out-of-pocket expenditure accounts for 21% of total health spending, well above the EU average of 14.5%,^10^^,^^11^ and 7.36% of households meet the WHO threshold for catastrophic dental expenditure.^12^ The number of registered dentists increased from 3946 in 1980 to 42,860 in 2024, representing a 986.2% increase,^13^ reaching a density of 85 per 100,000 (exceeding France, 69; Ireland, 46; and the United Kingdom, 51) and nearly triple the reference ratio employed in the comparative literature.^14^^,^^15^ Recent European evidence has also shown substantial heterogeneity in professional distribution: Fernández-Serrano et al. reported that maximum-to-minimum ratios for general dentists, dental specialists and specialist-to-dentist proportions across European countries differed by 2.5-, 5.7 and 4.1-fold, respectively, with additional intranational variation in France and Germany.^16^ This supports subnational workforce analyses rather than reliance on national averages alone. Yet 97% of professionals practice exclusively in the private sector,^17^^,^^18^ and utilisation remains among Europe's lowest (0.7 visits per inhabitant versus 1.6 in Germany and 2.8 in the Netherlands^15^^,^^19^), with 68% of Spaniards citing economic barriers.^20^

The public SNHS dental workforce represents fewer than 4% of all registered professionals,^17^^,^^18^ distributed across 19 governance arrangements.^21^^,^^22^ No centralised official data on its regional distribution exists.^17^^,^^23^

Using transparency requests under Law 19/2013^24^ submitted to all 19 administrations, this study aims to: (1) quantify the distribution of total, salaried public and privately accredited dentists across 17 Autonomous Communities and Ceuta and Melilla; (2) characterise inter-regional variation in public-sector dental capacity and structural dependence on private provision; and (3) assess the implications of this structural imbalance for evidence-based workforce planning and oral health equity, in alignment with the WHO Global Oral Health Action Plan 2023-2030^1^ and Sustainable Development Goal 3.^25^

## Material and methods

### Study design

Cross-sectional observational study with comparative analysis. Data as of 2024-2025. The study was designed to compare territorial workforce capacity at the latest available time point and was not designed to estimate temporal trends in public dental workforce development.

### Data sources

Structured transparency requests to all 19 administrations under Law 19/2013,^24^ supplemented by official professional registration data (Colegios Oficiales de Dentistas, 2024),^26^ INE population denominators and historical registered-dentist series,^13^ and Ministry of Health records.^27^^,^^28^ Workforce counts refer to professionals or posts reported by administrations, not full-time-equivalent staffing hours.

Galicia did not respond; data were obtained from the Ministry of Health statistical portal (2022). The Ministry of Health's central registry (REPS) was formally declared incomplete for dentistry under Article 18.1(d) of Law 19/2013,^23^^,^^24^ confirming the necessity of a bottom-up collection strategy. Full data collection procedure is described in Appendix S1.

### Models of provision

Three configurations were distinguished: (1) direct public provision (salaried SNHS dentists in primary care centres); (2) PADI-type mixed model (privately accredited dentists reimbursed by capitation for publicly funded programs, primarily paediatric dental care^29^ and, in Madrid, care for those aged over 80 years, operating in their own clinics); and (iii) intermediate models combining elements of both (Castilla y León, Castilla-La Mancha).^22^^,^^29^^,^^30^

### Primary variables

Total active registered dentists per 100,000 inhabitants; salaried public-sector dentists per 100,000 inhabitants; privately accredited dental providers per 100,000 inhabitants.

Derived indicators: (1) Public/total ratio (%): salaried public dentists as a proportion of total registered professionals per AC; (2) Structural Dependence Index (SDI): accredited private providers as a proportion of effective public-system workforce (salaried public + accredited private) × 100; (3) population load per public dentist. SDI was designed as a structural indicator of reliance on accredited private providers within publicly funded dental programmes. It does not measure service volume, provider availability, continuity of care, treatment completion or clinical outcomes.

Analysis. Descriptive statistics (mean, median, SD, coefficient of variation [CV]). Spearman correlation between total density and public/total ratio was used as an exploratory descriptive test. Given the small number of territorial units (n = 19), correlation results were interpreted descriptively rather than as confirmatory statistical evidence. Full variable definitions and statistical methods in Appendix S1.

### Ethics

This study used exclusively public aggregated information obtained through legally established transparency mechanisms. No individual-level data was collected. Ethics approval was not required.

## Results

Responses were obtained from 18 of 19 administrations. Galicia was the only AC that did not respond to any transparency request; data were obtained from the Ministry of Health statistical portal (2022). The Ministry of Health, when formally consulted, issued a decision of nonadmission under Article 18.1(d) of Law 19/2013,^24^ explicitly stating that the National Registry of Health Professionals (REPS) 'is not yet complete' for dentistry.

In 2024, Spain had 42,860 active registered dentists (84.68/100,000; CV 24.9%) (Table 1). Inter-regional variation was substantial, ranging from 133.53/100,000 in the Comunidad de Madrid to 46.17/100,000 in Castilla-La Mancha; a nearly three-fold difference. All 19 territories exceeded the reference ratio of approximately 29 dentists per 100,000 inhabitants employed in comparative dental workforce literature,^14^^,^^15^ with the national average standing at nearly 3 times that threshold.

**Table 1:** Total registered active dentists per 100,000 inhabitants by Autonomous Community (2024).

Autonomous community	Total dentists/100,000	Model
Comunidad de Madrid	133.53	Mixed
Galicia	87.39	Public
Asturias	86.78	Public
País Vasco	85.98	Mixed
Murcia	85.06	Mixed
C. Valenciana	85.02	Public
Cataluña	78.45	Public
Andalucía	77.45	Mixed
La Rioja	76.12	Public
Cantabria	73.14	Public
Baleares	73.13	Mixed
Aragón	71.42	Mixed
Canarias	69.61	Mixed
Castilla y León	65.77	Mixed
Extremadura	62.87	Mixed
Navarra	57.06	Mixed
Melilla	57.47	Mixed
Ceuta	52.69	Public
Castilla-La Mancha	46.17	Mixed
National average	84.68	-

*Source*: Colegios Oficiales de Dentistas / INE 2024. CV = coefficient of variation.

The public-sector dental workforce comprised 1685 salaried dentists, yielding a mean territorial public-sector density of 3.36 per 100,000 inhabitants and a mean territorial population load of 31,590 inhabitants per public dentist (Table 2). The public/total ratio ranged from 2.0% in the Comunidad de Madrid to 7.6% in Castilla-La Mancha, with a CV of 31.3%, exceeding inter-regional variation in total dentist density (CV 24.9%). No AC reached a public/total ratio above 8%, confirming that the overwhelming majority of the dental workforce operates outside the public system in every region. The contrast between total density (84.68/100,000) and mean territorial public-sector density (3.36/100,000) is consistent with a national public/total ratio of approximately 4%: roughly 96% of Spain's dental professionals operate outside the SNHS.

**Table 2:** Public-sector dentists per 100,000 inhabitants and public/total ratio by Autonomous Community (2024-2025).

Autonomous community	Public/100,000	Population load	Public/Total %	Model
Cataluña	5.17	19,353	6.6%	Public
Galicia	4.53	22,072	5.2%	Public
Murcia	4.22	23,715	5.0%	Mixed
Asturias	4.08	24,624	4.7%	Public
Cantabria	3.88	25,804	5.3%	Public
Canarias	3.66	27,302	5.3%	Mixed
Ceuta	3.61	27,812	6.9%	Public
Castilla-La Mancha	3.51	28,438	7.6%	Mixed
Extremadura	3.51	28,456	5.6%	Mixed
Castilla y León	3.08	29,099	4.7%	Mixed
La Rioja	3.08	32,418	4.0%	Public
Baleares	3.05	32,745	4.2%	Mixed
Andalucía	3.03	32,763	3.9%	Mixed
C. Valenciana	2.87	33,072	3.4%	Public
Madrid	2.70	34,836	2.0%	Mixed
Melilla	2.40	37,086	4.2%	Mixed
País Vasco	2.20	45,463	2.6%	Mixed
Aragón	2.14	46,690	3.0%	Mixed
Navarra	2.06	48,452	3.6%	Mixed
National mean	3.36	31,590	4.7%	-

*Source*: Autonomous Community responses to transparency requests, 2024-2025; Galicia: Ministry of Health 2022. CV = coefficient of variation; SDI = Structural Dependence Index.

A total of 6810 privately accredited dentists provided publicly funded dental care across 11 mixed-model ACs under PADI-type arrangements,^22^^,^^29^^,^^30^ receiving capitation fees and per-procedure reimbursements (Table 3). When included, the effective public-system workforce reached 8495 professionals (17.21/100,000), 5 times the public-only figure. Among territories with reported individual accredited-provider counts, the Structural Dependence Index (SDI) ranged from 0% in direct public-provision ACs (Asturias, Cantabria, Cataluña, Comunitat Valenciana, Galicia, La Rioja and Ceuta) to 95.4% in País Vasco. Melilla was not interpreted as a zero-dependence public model because its externalised service was reported through a company rather than as an individual accredited-provider count. Ten of eleven mixed-model ACs with individual accredited-provider data recorded SDI values above 80%, indicating that in these regions more than 4 in 5 dentists providing publicly funded care are privately accredited professionals.

**Table 3:** Accredited private dental providers, effective public-system capacity and Structural Dependence Index by Autonomous Community (2024-2025).

Autonomous community	Salaried public/100k	Accredited private/100k	Effective public/100k	SDI (%)	Model
País Vasco	2.20	45.83	48.03	95.4%	Mixed
Navarra	2.06	34.64	36.70	94.4%	Mixed
Aragón	2.14	20.31	22.45	90.5%	Mixed
Murcia	4.22	37.38	41.60	89.9%	Mixed
Andalucía	3.03	24.12	27.15	88.8%	Mixed
Castilla-La Mancha	3.51	24.95	28.46	87.7%	Mixed
Baleares	3.05	19.21	22.26	86.3%	Mixed
Madrid	2.70	16.82	19.52	86.2%	Mixed
Extremadura	3.51	21.56	25.07	86.0%	Mixed
Castilla y León	3.08	12.96	16.04	80.8%	Mixed
Canarias	3.66	5.49	9.15	60.0%	Mixed
Asturias	4.08	0	4.08	0%	Public
Cantabria	3.88	0	3.88	0%	Public
Cataluña	5.17	0	5.17	0%	Public
C. Valenciana	2.87	0	2.87	0%	Public
Galicia	4.53	0	4.53	0%	Public
La Rioja	3.08	0	3.08	0%	Public
Ceuta	3.61	0	3.61	0%	Public
Melilla	2.40	0	2.40	0%	Mixed*

⁎ Melilla externalises dental services to a company; number of individual professionals not reported. SDI = Structural Dependence Index = accredited private providers/(salaried public + accredited private) × 100. *Source*: Autonomous Community responses, 2024-2025.

**Fig. 1:**
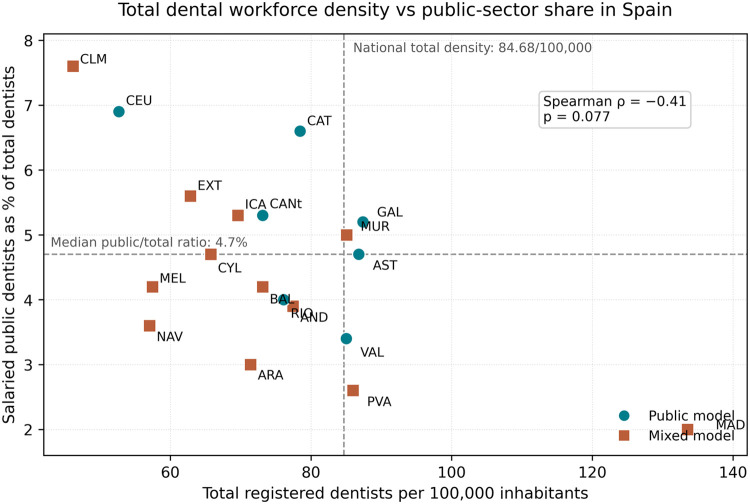
Scatter plot: Total registered dentists per 100,000 inhabitants (x-axis) vs Public/total ratio % (y-axis), with ACs labelled and color-coded by organisational model. Four quadrants defined by reference values (national total density: 84.68/100,000; median public/total ratio: 4.7%). Teal circles = public provision model; rust squares = mixed provision model. Source: Colegios Oficiales de Dentistas / INE 2024; transparency requests to Autonomous Community administrations, 2024-2025.

Figure 1 maps total dentist density against the public/total ratio for all 19 territories, suggesting a moderate inverse association that did not reach conventional statistical significance (Spearman ρ = –0.41; *P* = .077). This finding should be interpreted descriptively: high aggregate professional density did not consistently correspond to high public-sector capacity.

**Fig. 2:**
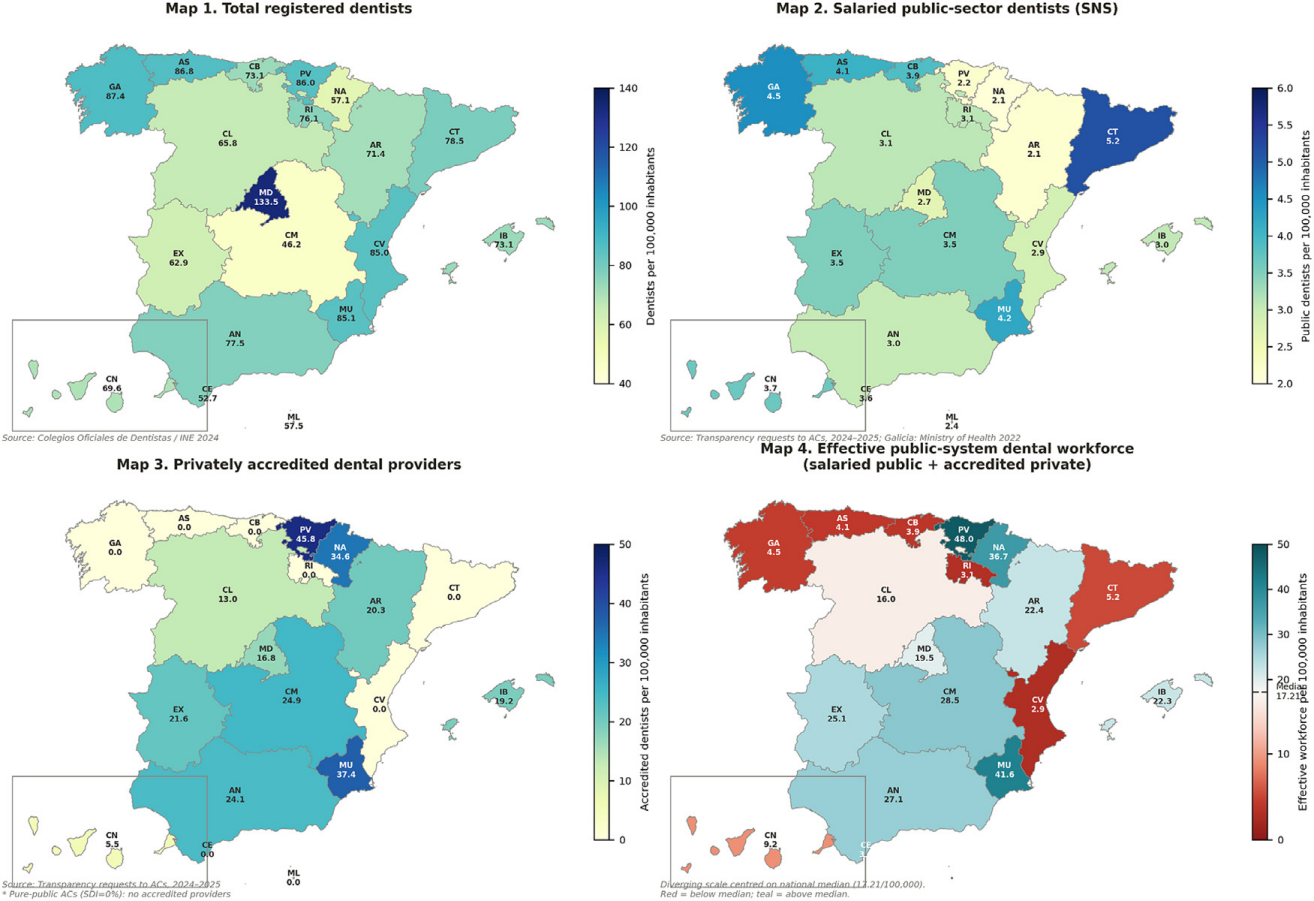
Territorial distribution of the dental workforce in Spain by provision type (2024-2025). Map 1: total registered dentists per 100,000 inhabitants. Map 2: salaried public-sector dentists per 100,000 inhabitants. Map 3: privately accredited dental providers per 100,000 inhabitants. Map 4: effective public-system dental workforce (salaried public + accredited private) per 100,000 inhabitants. Map 4 uses a diverging scale centered on the national reference value (17.21/100,000): red = below reference; teal = above reference. Source: Colegios Oficiales de Dentistas, INE and transparency requests to Autonomous Community administrations, 2024-2025. Map lines delineate study areas and do not necessarily depict accepted national boundaries.

Figure 2 presents choropleth maps of all 4 workforce dimensions, with Map 4 using a diverging scale to highlight the stark contrast between purely public ACs and high-SDI mixed-model regions.

## Discussion

**Fig. 3:**
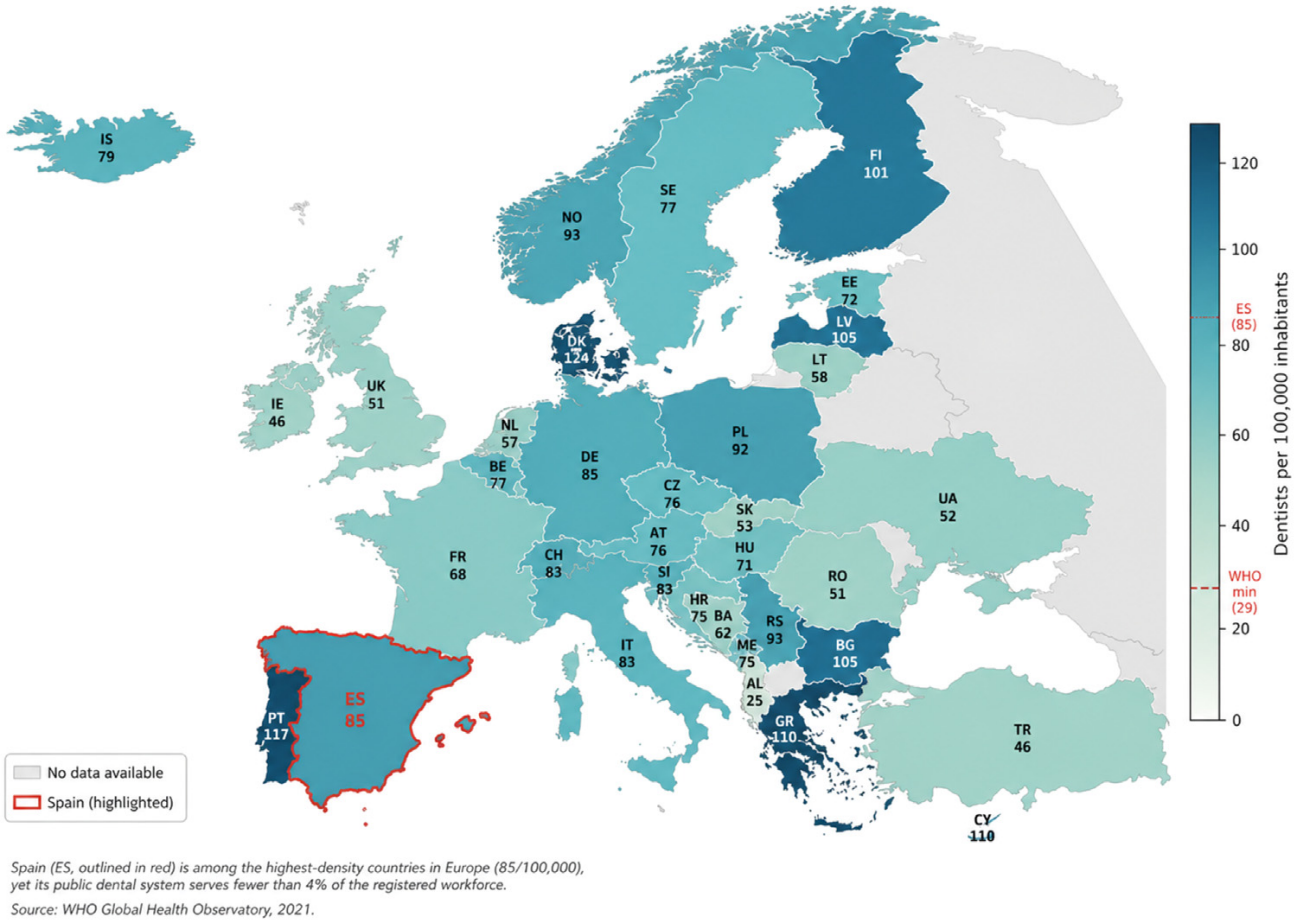
Dentist density per 100,000 inhabitants across European countries (WHO Global Health Observatory, 2021). Spain (ES, outlined in red) ranks among the highest-density countries in Europe (85/100,000), yet its public dental system encompasses fewer than 4% of the registered workforce. All European countries substantially exceed the reference density ratio employed in comparative dental workforce literature.^14^^,^^15^ Map lines delineate study areas and do not necessarily depict accepted national boundaries.

Spain registers 84.68 dentists per 100,000 inhabitants, a density among the highest in Europe as Figure 3 illustrates; exceeding France (68/100,000), the United Kingdom (51/100,000) and Ireland (46/100,000),^14^^,^^15^ and approaching levels observed in Greece (130/100,000) and Portugal (117/100,000).^31^ Spain's total dental workforce density exceeds that of France, Ireland and the United Kingdom^14^^,^^15^; however, this high professional availability does not translate into public-sector capacity. The SNHS comprises only 1685 salaried public dentists, with a mean territorial public-sector density of 3.36 per 100,000 and a mean territorial load of 31,590 inhabitants per public dentist, representing a small fraction of the total workforce.

This study confirms that the issue is not a shortage of professionals, but a structural imbalance in workforce allocation, with direct implications for oral health equity and system governance.^32^ Notably, the heterogeneity of public-sector provision exceeds that of total professional availability (CV 31.3% vs 24.9%), indicating that inter-regional disparities in access to public dental care are driven more by system organisation than by workforce distribution. These findings provide a workforce-architecture explanation for previously described territorial inequities in Spain’s publicly funded oral health provision.^22^^,^^33^ Effective access may depend not only on nationally defined entitlement, but also on regional workforce capacity and delivery model.

The Structural Dependence Index (SDI), defined as the proportion of effective public-system capacity constituted by privately accredited providers, offers a structural lens for health systems research in which public-private hybrid arrangements are pervasive. SDI should be interpreted as an indicator of governance architecture and potential dependence, not as a direct measure of effective clinical capacity. The finding that ten of eleven mixed-model ACs with individual accredited-provider data recorded SDI values above 80% reframes the conventional narrative of public-private partnership in oral health: in País Vasco (SDI 95.4%), Navarra (94.4%) and Aragón (90.5%), the direct public workforce functions less as an operational core than as an administrative and supervisory layer, with 9 in ten dentists providing publicly funded care operating through private capitation agreements. Privately accredited dentists are not stable members of the public workforce: they attend patients in their own clinics, are reimbursed through capitation fees and per-procedure tariffs and participate episodically. The effective workforce figure of 8495 therefore represents a conceptual approximation of potential rather than operational capacity. Furthermore, the financial viability of capitation models is not guaranteed: if reimbursement rates do not cover the cost of complex procedures (such as endodontics), providers may selectively deprioritise these treatments or, over time, withdraw from public programs altogether.^6^ This systemic fragility is distinct from the stability offered by salaried public employment.

Cross-referencing total density with the public/total ratio and SDI reveals instructive contrasts among extreme cases. The Comunidad de Madrid (133.53/100,000 total; public/total 2.0%; SDI 86.2%) combines the highest aggregate professional availability in Spain with near-maximum structural dependence on accredited private networks. País Vasco (85.98/100,000; public/total 2.6%; SDI 95.4%) presents an even more extreme dependence despite average total density. By contrast, Cataluña (public/total 6.6%; SDI 0%; 5.17 public/100,000) is the only purely public-model AC that achieves a population load below 20,000 inhabitants per public dentist. The WHO 1980 figure is retained only as a historical planning reference, not as a contemporary universal staffing standard.^34^ We did not identify an updated universally endorsed public-sector dental workforce ratio in current WHO, FDI, OECD or European Observatory guidance. Contemporary oral health workforce planning has shifted towards needs-based, skill-mix-oriented and FTE-adjusted approaches, which better account for population need, service design, productivity, working patterns, geographical distribution and the contribution of other oral health professionals.^35^, ^36^, ^37^ Galicia, Asturias and Cantabria also achieve comparable public-sector densities without any accredited network, consistent with evidence from other European purely public dental systems.^38^^,^^39^ The Comunitat Valenciana (SDI 0%; 2.87/100,000) illustrates the inverse: a purely public model serving Spain's fourth-largest population with the lowest effective public capacity in the country, confirming that the pure-public model does not automatically guarantee adequate staffing.^40^ These 4 cases demonstrate that neither aggregate density nor provision model alone determines public-system capacity; it is the combination of model, direct public staffing and SDI that defines potential public-system capacity.

A governance dimension compounds the workforce imbalance. Oral health legislation spans a spectrum from autonomous community laws (Cataluña Law 12/2020^41^; Madrid Law 7/2018^42^) through decrees, orders and resolutions to informational web content; Cataluña's law is the only statute that explicitly obliges the administration to conduct periodic workforce needs assessments. No AC updated its oral health legislation following the 2024 expansion of the common service portfolio,^9^ creating a temporal lag between national entitlement expansion and its normative transposition, with direct implications for implementation fidelity and resource mobilisation. This regulatory heterogeneity, documented here for the first time in a comparative framework, means that workforce planning obligations are legally binding in 1 AC and entirely absent in others.

The findings carry direct implications for oral health policy. First, the Ministry of Health has formally acknowledged its inability to produce centralised dental workforce data, as confirmed by a decision of nonadmission under Law 19/2013.^24^ At the same time, it administers €68 million annually in SNHS oral health transfers to ACs since 2022.^43^ This exposes a fundamental governance contradiction: coverage expansion cannot be planned, funded or monitored effectively without an information infrastructure capable of tracking the workforce expected to deliver it. By contrast, an equivalent transparency request regarding ophthalmologists was resolved with centralised figures, confirming that this is a specific, not a general, information governance gap. The establishment of a mandatory, standardised national dental workforce registry and a public oral health observatory, proposed repeatedly in the Spanish scientific literature since at least 1994^44^^,^^45^ and explicitly called for in the 2020 National Oral Health Survey,^46^ has become empirically urgent. This argument is consistent with recent IDJ evidence from the FDI Oral Health Observatory pilot, which demonstrated the feasibility of collecting standardised practice-based oral health data across countries using paired dentist-patient questionnaires and digital tools and emphasised the value of comparable oral health and service data for policy planning.^47^ Second, coverage expansion without commensurate direct public workforce development may increase reliance on privately accredited networks that, as this study demonstrates, constitute a fragile and conditionally reliable operational base. The WHO Global Oral Health Action Plan 2023-2030^1^ calls for integration of oral health into primary health care through public-sector workforce development: a standard that Spain's current architecture falls substantially short of meeting. The historical reference discussed above should be interpreted as a scale indicator rather than a current prescriptive standard; in the absence of updated Spanish public dental workforce benchmarks, it helps illustrate the magnitude of population loads currently borne by salaried public dentists. Under this framework, only Cataluña currently meets the historical minimum among purely public-model ACs; the eleven mixed-model ACs with reported individual accredited-provider counts surpass it exclusively by virtue of their accredited networks, a dependence that, as shown, is structurally fragile and analytically distinct from public workforce capacity.^48^

The present findings also suggest that Spain's expanded oral health portfolio may be implemented under structurally unequal conditions. Where public dental workforce capacity is very limited, the same legal entitlement may generate different practical results: some territories may be able to translate coverage expansion into accessible preventive and clinical services, whereas others may struggle to provide timely, continuous and equitable care. These findings provide a workforce-architecture explanation for previously described territorial inequities in Spain's publicly funded oral health provision.^22^^,^^33^ In broader health-policy literature, geographically contingent access to public services has been described as a ‘postcode lottery’, a term originating in UK debates on unequal NHS access and later used to denote place-based unfairness in health service provision.^49^ In the Spanish context, effective access may therefore depend not only on nationally defined entitlement, but also on regional workforce capacity and delivery model. Without minimum workforce planning standards and a national monitoring system, portfolio expansion risks increasing nominal coverage while leaving major differences in effective access unresolved.

Several limitations merit acknowledgement. Data collection relied on transparency requests; Galicia did not respond, necessitating use of 2022 Ministry data; Melilla externalises dental services to a single private company without reporting individual professional numbers. The study is cross-sectional and does not assess whether the imbalance between total and public dental workforce capacity is widening, stable or narrowing over time. Although the overall registered dental workforce increased by 986.2% between 1980 and 2024, comparable longitudinal data for salaried public dentists are not centrally available. Earlier partial approximations exist (for example, Llena Puy et al. reported public and mixed-model workforce resources using information collected in 2016) but their scope did not include all PADI-type regions and is not directly comparable with the present all-territory enumeration.^22^ Workforce counts refer to professionals, posts or programme-accredited providers, not full-time-equivalent staffing hours, part-time contracts, temporary appointments or actual clinical hours. Therefore, public workforce capacity may be overestimated where dentists work part-time and accredited-provider capacity may be overestimated where participation in publicly funded programmes is limited or intermittent. Accredited provider figures represent professionals registered in public programs, not volumes of patient contacts; effective utilisation of accredited capacity cannot be assessed from available data. The existence of accredited dentists in public programs does not imply continuous or comprehensive service delivery: capitation models may create implicit treatment selection if reimbursement does not cover the cost of complex procedures, a concern that warrants longitudinal monitoring. Future research integrating workforce data with service utilisation records and oral health outcomes would substantially advance the evidence base for workforce planning.

## Conclusions

Spain does not face a shortage of dental professionals. It faces a structural misalignment between a numerically abundant, almost exclusively private-sector workforce and a limited, unevenly monitored public dental system whose effective capacity depends, in eleven of nineteen territories, on a network of privately accredited professionals operating under capitation arrangements of uncertain long-term stability. This imbalance, documented here at autonomous community level using systematic transparency-request data, is not addressable through incremental coverage reforms that leave workforce architecture unchanged. Evidence-based oral health policy in Spain requires, as a first step, a centralised dental workforce registry and a national oral health observatory, followed by explicit public workforce planning targets calibrated to primary care standards.^50^^,^^51^ Without explicit workforce planning standards, expansion of publicly funded oral health coverage may increase formal entitlements without ensuring equivalent effective access across territories. Spain therefore illustrates a broader lesson for high-income countries with predominantly private dental markets: aggregate professional density is a poor proxy for public oral health system capacity.

## Ethics statement

This study used exclusively aggregated administrative and publicly available information obtained through legally established transparency mechanisms. No individual-level patient data were collected. Ethics approval was not required.

## Data availability

The aggregate, nonidentifiable dataset supporting the findings of this study is available from the corresponding author upon reasonable request. Data were obtained through formal transparency requests submitted to all Spanish regional health administrations and to the Spanish Ministry of Health under Law 19/2013 on Transparency, Access to Public Information and Good Governance. Requests for access will be considered when reasonable and scientifically justified.

## Declaration of generative AI and AI-assisted technologies in the manuscript preparation process

During manuscript preparation, ChatGPT (OpenAI) was used to support English-language editing, improve clarity and consistency of terminology and assist with formatting of editorial text. It was not used to collect data, generate the dataset, perform statistical analyses, create or modify figures, select results, draw conclusions or make authorial decisions. All AI-assisted text was reviewed, edited and verified by the authors, who take full responsibility for the content of the submitted manuscript.

## Funding

This research did not receive any specific grant from funding agencies in the public, commercial or not-for-profit sectors.

## Author contributions

Manuel Jesús Enciso Ripoll conceived the study, compiled the dataset, performed the analysis, interpreted the findings and drafted the manuscript. José Enrique Iranzo Cortés contributed to study design, interpretation of findings and critical revision of the manuscript. Manuel Bravo Pérez contributed to interpretation of findings, oral epidemiology framing and critical revision of the manuscript. Francisco Javier Silvestre Donat contributed to study design, interpretation of findings and critical revision of the manuscript. All authors approved the final version of the manuscript and agree to be accountable for all aspects of the work.

## Conflicts of interest

None disclosed.

## Appendix Supplementary materials

Image, application 1

## Footnotes

Supplementary material associated with this article can be found in the online version at doi:10.1016/j.identj.2026.109687.
