# Supplementary material for: Public Dental Workforce Capacity and Inequality in Spain
Source: Int Dent J. 2026 Jun 18;76(4):109687. doi: 10.1016/j.identj.2026.109687 (PMC13334410; doi:10.1016/j.identj.2026.109687)
Supplement: Supplementary file 1 [file mmc1.docx]

**Public dental workforce capacity and inequality in Spain**

**Appendix S1. Data collection procedure, variable definitions and statistical analysis**

Supplementary material for: Public dental workforce capacity and inequality in Spain

**1. Study setting and units of analysis**

The unit of analysis was the autonomous community or autonomous city. The study covered all 17 Spanish Autonomous Communities and the Autonomous Cities of Ceuta and Melilla. These 19 territorial administrations are responsible for the organization and delivery of publicly funded health services within Spain's decentralized National Health System.

**2. Data collection through transparency requests**

Administrative workforce information was obtained through formal transparency requests submitted to the health administrations of all 17 Autonomous Communities, the two Autonomous Cities and the Spanish Ministry of Health under Law 19/2013 on Transparency, Access to Public Information and Good Governance. The requests asked for the latest available counts of salaried public dentists working in the public health system and, where applicable, dentists accredited to provide publicly funded dental care through mixed or PADI-type arrangements.

**3. Supplementary official data sources**

Transparency responses were supplemented with official professional registration data from the Colegios Oficiales de Dentistas, official population denominators from the Instituto Nacional de Estadística (INE), and Ministry of Health records. Galicia did not respond to the transparency request; therefore, the latest available Ministry of Health statistical data were used for that territory. For Melilla, the externalized dental service model did not provide an individual professional count for accredited providers.

**4. Workforce categories**

Three workforce layers were distinguished. Total dentists referred to active registered dentists by territory. Salaried public dentists referred to dentists directly employed by the public health system. Accredited private providers referred to private dentists authorized or contracted to provide publicly funded dental care under mixed or PADI-type arrangements. Accredited providers were treated as part of effective publicly funded capacity, but not as direct public workforce.

**5. Provision model classification**

Territories were classified according to their documented organizational model: direct public provision, mixed provision or PADI-type mixed provision. Direct public models rely mainly on salaried public teams. Mixed and PADI-type models include substantial participation of privately accredited providers in publicly funded oral health services, particularly pediatric care. This classification was used for descriptive and interpretive purposes and does not assume that all accredited providers deliver equivalent volumes of care.

**6. Indicators and formulas**

Public dentist density was calculated as salaried public dentists per 100,000 inhabitants. Population load was calculated as inhabitants divided by salaried public dentists. The public/total ratio was calculated as salaried public dentists divided by total active registered dentists, multiplied by 100. Effective public-system workforce was calculated as salaried public dentists plus accredited private providers. The Structural Dependence Index (SDI) was calculated as accredited private providers divided by effective public-system workforce, multiplied by 100.

**7. Statistical analysis**

The analysis was descriptive and comparative. For each territory, workforce densities, population loads, public/total ratios and SDI values were calculated. Inter-territorial variation was summarized using means, medians, standard deviations and coefficients of variation where appropriate. Spearman correlation was used to assess the association between total dentist density and the public/total ratio. No causal inference regarding service utilization or oral health outcomes was attempted.

**8. Interpretation of accredited-provider capacity**

Accredited private providers should not be interpreted as equivalent to salaried public employees. They may provide publicly funded services in their own clinics, under capitation or mixed reimbursement arrangements, and their participation may vary by territory and program. The effective public-system workforce indicator therefore represents a conceptual approximation of potential publicly funded capacity rather than a direct measure of operational full-time-equivalent public staffing.

**9. Data limitations**

Administrative responses may differ in classification criteria, reference dates and reporting practices. Some territories reported post counts, whereas others reported professionals or program-accredited providers. The analysis does not measure clinical activity, waiting times, patient-level access, treatment completion or oral health outcomes. These limitations justify cautious interpretation and support the need for a centralized, standardized public oral health workforce registry. Full-time-equivalent staffing hours, part-time contracts, temporary appointments and actual clinical hours were not available in a standardized or comparable format across autonomous administrations.

**10. Reproducibility statement**

All derived indicators were calculated from aggregated territorial counts and population denominators. The aggregate, non-identifiable dataset is available from the corresponding author upon reasonable request, subject to reasonable scientific justification and compatibility with the conditions under which the administrative information was obtained.

**Supplementary Table S1. Variable definitions**

| Variable or indicator | Definition | Interpretation |
| --- | --- | --- |
| Total dentists/100,000 | Active registered dentists divided by population, multiplied by 100,000. | Overall professional supply, not public-system capacity. |
| Salaried public dentists/100,000 | Dentists directly employed by the public health system divided by population, multiplied by 100,000. | Direct public dental workforce capacity. |
| Population load | Population divided by salaried public dentists. | Number of inhabitants served per public dentist. |
| Public/total ratio | Salaried public dentists divided by total active registered dentists, multiplied by 100. | Share of the total dental workforce located in direct public employment. |
| Accredited private providers/100,000 | Privately accredited providers participating in publicly funded programmes divided by population, multiplied by 100,000. | Contracted or mixed public-programme capacity. |
| Effective public-system workforce | Salaried public dentists plus accredited private providers. | Approximation of potential publicly funded dental workforce capacity. |
| Structural Dependence Index (SDI) | Accredited private providers divided by effective public-system workforce, multiplied by 100. | Dependence of publicly funded provision on accredited private providers. |
